# General Observations

**Published:** 1879-08

**Authors:** 


*General Observations.*—The above treatment should be persevered in for some hours. Persons have been restored after many hours of continued effort. The struggle should not be given up until a physician pronounces that death has unmistakeably occurred.

